# First report of *bla*_GES-1_ in *Proteus mirabilis* clinical isolates

**DOI:** 10.1590/0037-8682-0864-2020

**Published:** 2021-03-22

**Authors:** Elizabeth Maria Bispo Beltrão, Érica Maria de Oliveira, Ana Catarina de Souza Lopes

**Affiliations:** 1 Universidade Federal de Pernambuco, Departamento de Medicina Tropical, Recife, PE, Brasil.

**Keywords:** Proteus mirabilis, *bla*_GES_, *bla*_NDM_

## Abstract

*Proteus mirabilis* is one of the main pathogens causing urinary tract infections and sepsis. To our knowledge, this is the first report of a *P. mirabilis* hosting *bla*_GES_. The presence of these genes was determined using PCR and sequencing. We identified the presence of *bla*_GES-1_ in all three isolates. In addition, we identified the *bla*_KPC-2_ and *bla*_NDM-1_ genes in the two strains. These data emphasize the importance of monitoring and surveillance of all enterobacteria. The circulation of *P. mirabilis* strains carrying *bla*_GES-1_ constitutes a new scenario of resistance in this species and should be an epidemiological alert for global health.

## INTRODUCTION

*Proteus spp*. are gram-negative bacilli belonging to the order *Enterobacterales*. The genus contains six species: *Proteus vulgaris*, *Proteus mirabilis*, *Proteus myxofaciens*, *Proteus cibarius*, *Proteus penneri*, and *Proteus hauseri*. *P. mirabilis*, *P. vulgaris*, and *P. penneri* are commonly described as opportunistic pathogens^1^.

*Proteus* strains with moderate resistance to imipenem are frequent, with evidence that this resistance is due to changes in the penicillin-binding protein (PBP2). In addition, *Proteus* can acquire resistance to several antimicrobial groups because of the presence of resistance genes such as *bla*_OXA-58_, *bla*_OXA-48_, *bla*_OXA-23_, *bla*_NDM-1_, *bla*_VIM-1_, *bla*_IMP_, *bla*_KPC-2_, *bla*_ACC-1_, *bla*_FOX_, *bla*_CMY-2_, *bla*_DHA-1_, *bla*_VEB-6_, *bla*_PER-2_, *bla*_CTX-M_, and *bla*_TEM_ ^1^.

*Proteus mirabilis* is a bacterium frequently associated with urinary tract infections (UTI), especially in patients who have been using a urinary catheter for a long time. In addition, it has intrinsic resistance to polymyxins, nitrofurantoin, and tigecycline, which makes it difficult to treat infections caused by this bacterium^2^. In this context, infections by *P. mirabilis* presenting acquired resistance to beta-lactams are considered challenging issues for antimicrobial therapy.

Class A extended-spectrum beta-lactamases (ESBL) are determinants of acquired resistance that have a high clinical impact^3^. The emergence of ESBLs, such as GES, has become an emerging public health problem, along with the NDM and KPC carbapenemases. GES confers resistance to all extended-spectrum cephalosporins and adversely impacts the efficiency of ceftazidime, and KPC and NDM to all beta-lactams. 

The current literature describes the presence of *bla*_GES_ in several bacterial species and in several countries distributed across all continents. However, the presence of this gene in *Proteus* has not been extensively investigated. We searched worldwide databases (Pubmed/NCBI, scielo, Google scholar, and nucleotide sequences deposited on the Nucleotide/NCBI platform) for manuscripts that reported the presence of *Proteus* isolates carrying *bla*_GES_ (keywords for the searches: “*Proteus*” and “*bla*_GES_,” “*Proteus*” and “GES,” “*Proteus mirabilis*” and “*bla*_GES_”). Unexpectedly, none of the manuscripts reported the presence of *bla*_GES_ in *P. mirabilis* or any species belonging to the genus *Proteus*. To the best of our knowledge, this is the first case report of an isolate of *P. mirabilis* carrying *bla*_GES_.

## CASE REPORT

Three bacterial strains isolated from three patients from a public hospital in Recife-PE, Brazil, were analyzed. The first isolate (P5-A2) was obtained from a female patient with urinary infection and was admitted to the surgical clinic. The second isolate (P15-A2) was obtained from a female patient with urinary tract infection and was admitted to the cardiology department. The third isolate (P44-A2) was obtained from a female patient with a sample collected from the catheter tip, who was admitted to the intensive care unit (ICU).

Biochemical identification and susceptibility to different classes of antimicrobials were performed using the automated system Bactec 9120/Phoenix-BD. Susceptibility interpretation was performed according to the BrCast^4^. The presence of *bla*_GES_, *bla*_NDM_, and *bla*_KPC_ was determined by PCR using specific primers^5^. The PCR products were sequenced using Sanger sequencing. The nucleotide sequences were analyzed using BLAST (http://www.ncbi.nlm.nih.gov/), Clustal W of the European Bioinformatics Institute (http://www.cbi.ac.uk/), and Bioedit v7. The nucleotide sequences were deposited in GenBank under the accession numbers MW527065 and MW554921.

The three isolates showed multidrug resistance and phenotypic indication for the production of ESBL and/or carbapenemases, presenting resistance to several antimicrobials (Table 1). Only the isolate P44-A2 showed resistance to carbapenems, imipenem, and meropenem (Table 1). Sequencing analysis revealed the presence of *bla*_GES-1_ variants in the three isolates (P5-A2, P15-A2, and P44-A2). In addition to the presence of *bla*_GES-1_, we also identified the presence of *bla*_KPC-2_ and *bla*_NDM-1_ genes in isolates P15-A2 and P44-A2, respectively.

**TABLE 1:** Profile of *Proteus mirabilis* isolates analyzed in the study.

Id.	Harvest date (ddmmyyyy)	Sector	Clinical samle	Resistance rofile
**P5-A2**	24082017	Surgical clinic	Urine	Amoxicillin-clavulanate (MIC>168)
				Amicillin (MIC>16)
				Aztreonam
				Cehalothin (MIC>16)
				Cefeime (MIC>16)
				Ceftazidime
				Ceftriaxone (MIC>32)
				Cefuroxime (MIC>16)
				Cirofloxacin (MIC>2)
				Gentamicin (MIC>8)
				Levofloxacin (MIC>4)
				Trimethorim-sulfamethoxazole (MIC>476)
**P15-A2**	05012017	Cardiology	Urine	Amikacin (MIC>32)
				Amoxicillin-clavulanate (MIC>168)
				Amicillin (MIC>16)
				Aztreonam
				Cehalothin (MIC>16)
				Cefeime (MIC>16)
				Ceftazidime
				Ceftriaxone (MIC1=6)
				Cefuroxime (MIC>16)
				Cirofloxacin (MIC>2)
				Levofloxacin (MIC>4)
				Nitrofurantoin (MIC>64)
				Pieracillin-tazobactam (MIC>644
				Trimethorim-sulfamethoxazole (MIC>476)
**P44-A2**	03052018	ICU	Catheter ti	Amikacin (MIC=32)
				Amoxicillin-clavulanate (MIC>168)
				Amicillin (MIC>16)
				Aztreonam
				Cefeime (MIC>16)
				Cefoxitin (MIC>16)
				Ceftazidime (MIC>16)
				Ceftriaxone (MIC>32)
				Cefuroxime (MIC>16)
				Cirofloxacin (MIC>2)
				Gentamicin (MIC>8)
				Imienem (MIC>8)
				Levofloxacin (MIC>4)
				Meroenem (MIC=8)
				Nitrofurantoin (MIC>64)
				Pieracillin-tazobactam (MIC>644)
				Trimethorim-sulfamethoxazole (MIC>476)

**ICU:** intensive care unit; **MIC:** minimum inhibitory concentration; **Id:** identification.

## DISCUSSION

The presence of important resistance genes occurs less frequently in *Proteus* ^6^, since there are few reports of β-lactamases in this species when compared to other species of the Enterobacterales order. However, the presence of ESBL in *Proteus* was more frequent^7^.

GES enzymes were initially described in *Klebsiella pneumoniae* in France using a rectal swab from a one-month-old girl who was hospitalized in French Guiana^7^. Since then, *bla*_GES_ has been reported in several species and in several countries, including Brazil, most frequently in non-fermenting bacilli such as *Pseudomonas aeruginosa*, *Acinetobacter baumannii*, and enterobacteria such as *K. pneumoniae* ^8^ ^-^ ^10^. GES is part of a group of ESBL and carbapenemases, comprising 43 variants (http://bldb.eu/BLDB.php?prot=A#GES) that have been found in several species of gram-negative bacteria^3^. In the three isolates investigated, a minimum inhibitory concentration (MIC) value greater than 16 for ceftazidime was observed. GES-1 can hydrolyze aztreonam, amoxicillin, and mainly ceftazidime with a very high MIC value (MIC = 4 to MIC = 128)^7^.

The genes investigated in the present study were obtained from the bacterial genome and may contain plasmids that have not yet been identified. *bla*_GES_ is constantly found in plasmids, but it can be present in the chromosome and in conjugative or non-conjugative plasmids, such as IncP, ColE1, IncQ, and IncF, as well as in non-typable plasmids^11^ ^-^ ^12^.

The presence of *P. mirabilis* carrying *bla*_GES-1_ is worrying because this gene encodes the ESBL enzyme and shows resistance to a wide range of β-lactams, such as resistance to 3rd generation (ceftazidime, cefotaxime, cefoxitin) and 4th generation cephalosporins (cefepime) and monobactams (Aztreonam)^13^. In addition, within the *Proteus* genus, there are no reports of this pathogen carrying *bla*_GES_. These data emphasize the importance of monitoring and surveillance of all enterobacteria. The circulation of *P. mirabilis* strains carrying *bla*_GES-1_ constitutes a new scenario of resistance in this species and should be an epidemiological alert for global health.

**Ethical Approval:** The study was approved by the ethics committee for research involving human beings (CEP/Plataforma Brasil) and registered under CAAE number 99147818.6.0000.5208.
